# Shrink pattern of breast cancer after neoadjuvant chemotherapy and its correlation with clinical pathological factors

**DOI:** 10.1186/1477-7819-11-166

**Published:** 2013-07-24

**Authors:** Shushu Wang, Yi Zhang, Xinhua Yang, Linjun Fan, Xiaowei Qi, Qingqiu Chen, Jun Jiang

**Affiliations:** 1 Breast Disease Center, Southwest Hospital, Third Military Medical University, 29 Gaotanyan Street, Chongqing 400038, China

**Keywords:** Breast cancer, Breast conservation surgery, Neoadjuvant chemotherapy, Residual tumor, Subserial section

## Abstract

**Background:**

Breast conservation therapy (BCS) after neoadjuvant chemotherapy (NCT) can improve patients’ quality of life. Currently used intraoperative examination for negative margins may not be sufficient to detect microresidual foci, which are a risk factor for local recurrence. This study was conducted to investigate the shrinking pattern of breast cancer and residual tumors as a risk factor for BCS after NCT.

**Methods:**

Ninety women with stage II or III invasive ductal carcinoma who achieved partial response after NCT with paclitaxel and epirubicin were enrolled. All patients had undergone modified radical mastectomy. One-half of the surgical specimens were subjected to subserial sectioning. Pathological changes of tumor bed and pericancerous tissues were examined with an optical microscope. The levels of estrogen receptors, progesterone receptors and HER2 were analyzed by immnohistochemical staining.

**Results:**

The residual tumors were classified into three types according to their microscopic morphology: solitary lesion, multifocal and patchlike lesions, and main residual tumor with satellite lesions. Type I residual tumors were found in 55 patients (61%), type II in 30 patients (33%) and type III in 5 patients (6%). Types II and III were often associated with larger primary tumors. The types of residual tumors were not correlated with the status of hormone receptors or HER2.

**Conclusion:**

Three types of residual tumors were observed after NCT. The solitary residual tumor is most common, but main residual tumors with satellite lesions are most likely to cause local recurrence after BCS. Subserial sectioning would improve the identification of microfoci and patient survival after BCS.

## Background

Breast conservation therapy (BCS) can improve patients’ quality of life. Large-scale, multicenter, randomized controlled trials have shown no significant difference in recurrence-free survival and overall survival (OS) between patients who have undergone BCS and those who have had radical mastectomy [1] in early breast cancer. BCS entails a local recurrence rate of 10% in early breast cancer, so there is an emphasis on ways of decreasing local recurrence rate after BCS.

Neoadjuvant chemotherapy (NCT) can make more patients suitable for BCS by downstaging primary tumors [2,3]. However, NCT-downstaged tumors have a higher local recurrence rate than the primary tumors suitable for BCS, which may be a result of incomplete removal of cancer tissues [4].

Intraoperative frozen section examination is used to ensure negative margins. Some patients still develop local recurrence after BCS, however, despite negative margins reached. Recurrence is often near the site of the original tumor. Concentric or patchlike residual tumors remain at cancer sites after NCT. The patchlike residual cancer is one of the major factors underlying local recurrence after BCS [5]. Conventional pathological methods cannot reveal the general state of a tumor shrinking pattern and may miss microfoci, owing to specimen limitation. In this study, subserial sectioning was used to check the shrinking pattern and identify residual tumors after NCT. Our study should provide information that will help reduce local recurrence and enhance the survival of patients who have undergone BCS.

## Methods

### Patients and general protocols

All patients were enrolled and treated at the Breast Disease Center of the Southwest Hospital between 1 September 2007 and 1 October 2009. We enrolled 90 patients with stage II or III breast cancer. All patients were females from age 28 to about 70 yr (mean: 48.99 yr) (Table 1). Cancers were confirmed by core needle biopsy before NCT. Before chemotherapy or surgery, color Doppler ultrasound and X-ray examinations were performed to evaluate tumor size. Patients were given a paclitaxel and epirubicin (TE) regimen (paclitaxel 175 mg/m^2^, epirubicin HCl 80 mg/m^2^ iv, 21 d per round) for three to eight rounds. Routine blood, liver, kidney and heart function was examined before each round of treatment. Prior to surgery, ultrasound and X-ray examinations were performed again, the largest tumor diameter after chemotherapy was measured and lesion calcification and type or any small satellite lesions near the main mass were observed. All protocols were approved by the Ethics Committee of Third Military Medical University, and every patient signed the informed consent form.

**Table 1:** Patients’ clinical characteristics

**Characteristics**	**No. of patients**	**%**
Age (yr)		
≤ 40	18	20.0
41 to 60	65	72.2
>60	7	7.8
Chemotherapy (cycle)		
≤ 4	80	88.9
>4	10	11.1
T staging		
T1	14	15.6
T2	63	70.0
T3	13	14.4

### Inclusion criteria

1. Stage II (diameter of 2 to 5 cm) or stage III (>5 cm without involvement of skin or chest wall) invasive ductal carcinoma confirmed by core needle biopsy.

2. No involvement of lymph node determined by axillary dissection.

3. Color ultrasound and X-ray examinations showing that the primary tumor was a solitary lesion.

4. Response Evaluation Criteria in Solid Tumors (RECIST) [6] was used and partial response reached.

5. No chemotherapy, radiotherapy, hormonal therapy or any resection biopsy before surgery.

6. Not fit for BCS or patient had personally asked for modified radical mastectomy.

### Exclusion criteria

1. Patients diagnosed with primary cancer with multiple lesions.

2. Partial response not achieved after chemotherapy.

3. Clinically complete relief obtained after chemotherapy.

4. Patients undergoing BCS.

5. Tumor invasion of chest and skin.

6. Patients with stage I or IV breast cancer.

7. Patients who had undergone chemotherapy, radiotherapy, endocrine therapy and resection biopsy previously.

### Subserial section of whole breast

Subserial sections of the whole breast were prepared from the specimens. The sample was put on a board with the cutting surface facing downward, surrounding fat tissue and aponeurosis were cut off and the spindle-shaped skin border was sutured with silk and fixed to the board to prevent skin retraction. The specimen was stored in a −20°C ice box for 24 h, then parallel lines were drawn every 5 mm from the cutting edge to the margin of the half breast sample. Using these lines, 5-mm tissue pieces were cut, retrimmed to remove surrounding fat tissue and numbered sequentially. The tissue pieces were fixed in 10% formalin for 48 h, dehydrated with acetone and anhydrous alcohol for 12 and 24 h separately, soaked in chloroform and xylene separately for 24 h each, wax-dipped for 24 hours (melting point, 56°C to 58°C) and embedded in paraffin. Eight- to twelve-micrometer sections were cut using a Leica RM2016 slicer (Leica Biosystems, Nussloch, Germany) and stained with hematoxylin and eosin.

Histological evaluation was done according to the method of Miller and Payne [7]. The shape, distribution and size of the residual lesion, as well as hyperplasia of fibrous tissue, lymphocyte infiltration and change of pericancerous tissue, were recorded.

### Detection of estrogen receptors, progesterone receptors and HER2

The levels of estrogen receptors (ERs), progesterone receptors (PRs) and HER2 in preoperative core needle biopsy tissues and final surgical specimens were examined by immunohistochemical staining. ER and PR levels were determined by Allred score [8], which is based on the percentage of tumor cells (PS), intensity of the staining (IS) and total score (TS = PS + IS). PS represented the estimated proportion of tumor cells staining positive as follows: 0 (none), 1 (tumor cells staining positive < 1%), 2 (tumor cells staining positive between 1% and 10%), 3 (tumor cells staining positive between 10% and 33%), 4 (proportion of tumor cells staining positive between 33% and 67%) and 5 (proportion of tumor cells staining positive >67%). IS represented the average intensity of the positive cells as follows: 0 (none), 1 (weak), 2 (intermediate) and 3 (strong). PS and IS were then added to obtain TS as follows: negative (0 to about 2), 1+ (3 to about 4), 2+ (5 to about 6), and 3+ (7 to about 8).

A herceptin test [9] was used to detect HER2 levels. It was scored according to cell membrane staining, indicated as 3+ (strong, complete membrane staining in >10% of tumor cells), 2+ (weak to moderate, complete membrane staining in >10% of the tumor cells), 1+ (faint membrane staining involving only a portion of the membrane in >10% of tumor cells) or 0 (no staining or faint staining in < 10% of the tumor cells).

### Statistical analysis

SPSS version 13.0 software (SPSS, Inc, Chicago, IL, USA) was used for data analysis. A paired test was applied for matched data and *χ*^2^ and nonparametric tests were done for other data, with *P* < 0.05 regarded as significant.

## Results

### Light microscopic observation of pathological changes in tumor bed and pericancerous tissues after NCT

**Figure 1:**
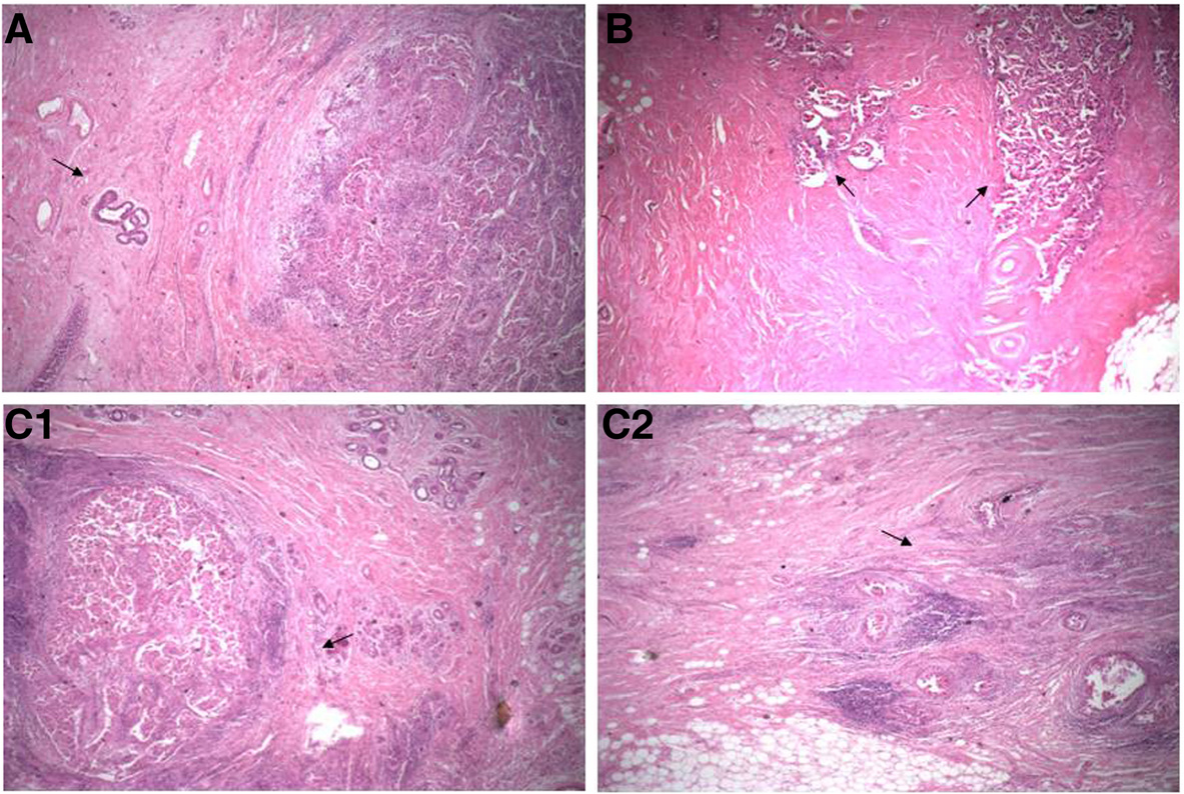
**Three types of residual cancers. (A)** Type I residual cancer. Solitary residual breast cancer can be seen (arrow). There is a large quantity of lymphocyte infiltration in the margin of the cancer lesion. There are no residual cancer cells, cancer cell clumps or satellite lesions near the cancer lesion. **(B)** Type II residual cancer. Two residual cancer lesions can be seen (arrows), and there is fibrotic change between the lesions. **(C)** Type III residual tumor. Solitary residual lesion (C1, arrow) and satellite lesion 1.5 cm away from the main lesion (C2, arrow) can be seen (hematoxylin and eosin stain; original magnification, ×40).

There were fibroplasias and lymphocyte infiltration to different degrees near the degenerated cancer site and small blood vessels (Figure 1). Histological classification after chemotherapy showed grade I in 4 cases, grade II in 26, grade III in 51 and grade IV in 10. Twenty-four cases with severe lymphocyte infiltration and sixty-six with light to moderate lymphocyte infiltration were found. Five cases had an intraductal carcinoma component.

### Morphological typing of residual breast cancer after NCT

There were three microscopic morphological types of residual breast cancer after NCT. Type I comprised solitary lesions in the fibrotic tumor bed with extensive lymphocyte infiltration near the cancer lesion (Figure 1A). Type II involved fibrotic changes in the tumor bed, which divided the residual cancer structure into several lesions with irregular shapes and patchlike shapes of different sizes (Figure 1B). Type III had a main cancer lesion and one or two small satellite lesions at least 1.0 cm away from it (10 mm to about 25 mm) visible in the fibrotic cancer bed with or without scattered cancer cell structures around the cancer lesion (Figures 1C1 and 1C2). Among the 90 patients, type I residual cancer was found in 55 (61%), type II in 30 (33%) and type III in 5 (6%). Among the patients with type III residual cancer, four had one satellite lesion and one had two satellite lesions.

### Mammary X-ray features of calcification and satellite lesions of breast cancer after chemotherapy

Mammary X-ray examination after NCT showed calcification in nine cases. Three had clusterlike granules around the tumor, one had linelike calcification distributed along the breast ducts and five showed large calcification particles located within the tumor. No satellite lesions were found by X-ray examination.

### Maximum tumor diameters measured by ultrasonography and pathological examination

Tumor size was measured after NCT using color Doppler ultrasound. The maximum diameter was 8 to 49 mm (mean, 19.2 mm). Pathological observation of postoperative subserial sections of whole breasts showed that the maximum tumor diameter was 5 to 51 mm (mean, 21.7 mm). The paired *t*-test showed no statistical difference in stage I breast cancer (*P* > 0.05), but significant differences in stages II and III breast cancer (*P* < 0.05) between the two examination methods were found.

### Relationship between types of residual tumor, age and menstrual status

The proportions of three age groups (≤ 40, 41 to 60 and >60 years of age) were 21.8%, 70.9% and 7.3%, respectively, for type I residual tumors; 13.3%, 80.0% and 6.7%, respectively, for type II residual tumors; and 20.0%, 60.0% and 20.0%, respectively, for type III residual tumors. There was no statistically significant difference among the three types (*P* > 0.05). The proportions of postmenopausal patients in the three types were 20.0%, 56.7% and 20.0%, respectively, which had no statistical significance (Table 2).

**Table 2:** Relationship between types of residual tumor with age and menstrual status

**Types**	***n***	**Age (yr) (%)**			**Menstrual status (%)**	
		**≤ 40**	**41 to 60**	**>60**	**Postmenopausal**	**Premenopausal**
Type I	55	12 (21.8)	39 (70.9)	4 (7.3)	22 (20.0)	33 (80.0)
Type II	30	4 (13.3)	24 (80.0)	2 (6.7)	17 (56.7)	13 (43.3)
Type III	5	1 (20.0)	3 (60.0)	1 (20.0)	1 (20.0)	4 (80.0)
*χ*^2^					3.465	
*P* value^a^		0.393			0.177	

^a^Kruskal-Wallis test and *χ*^2^ test.

### Relationship between types of residual tumor and primary tumor T stage

For type I residual tumors, the proportions of T1, T2 and T3 primary tumors were 20.0%, 76.4% and 3.6%, respectively. There were 10.0%, 56.7% and 33.3% of type II residual tumors resulting from T1, T2, and T3 primary tumors, respectively, whereas 20.0%, 60.0% and 20.0% of type III residual tumors came from T1, T2, and T3 primary tumors after NCT. The primary tumor stage distribution among the three types of residual cancer was statistically different (*P* < 0.05). Type II and type III residual tumors had higher occurrences in patients with larger primary tumors.

### Relationship between types of residual tumor and levels of ER, PR and HER2

The positive rates of ER, PR and HER2 before and after NCT were not statistically different among patients with three types of residual tumors (*P* > 0.05) (Table 3). The changes in expression levels of ER, PR and HER2 among three types of residual cancers also were not statistically different (*P* > 0.05) (Table 4).

**Table 3:** Relationship between types of residual tumor and expression of ER^a^, PR^a^ and HER2^b^

**Types**	**No. of ER (%) prior to NCT**				**No. of ER (%) after NCT**			
	**-**	**+ to about +++**	***χ***^**2**^	***P *value**	**-**	**+ to about +++**	***χ***^**2**^	***P *value**
Type I	21 (38.2)	34 (61.8)	2.032	0.362	22 (40.0)	33 (60.0)	0.378	0.828
Type II	7 (23.3)	23 (76.7)			10 (33.3)	20 (66.7)		
Type III	2 (40.0)	3 (60.0)			2 (40.0)	3 (60.0)		
	**No. of PR (%) prior to NCT**				**No. of PR (%) after NCT**			
	**-**	**+ to about +++**	***χ***^**2**^	***P *value**	**-**	**+ to about +++**	***χ***^**2**^	***P *value**
Type I	13 (23.6)	42 (76.4)	0.499	0.779	13 (23.6)	42 (76.4)	0.153	0.926
Type II	9 (30.0)	21 (70.0)			8 (26.7)	22 (73.3)		
Type III	1 (20.0)	4 (80.0)			1 (20.0)	4 (80.0)		
	**No. of HER2 (%) before NCT**				**No. of HER2 (%) after NCT**			
	**– to about +**	**++ to about ++++**	***χ***^**2**^	***P *value**	**– to about +**	**++ to about +++**	***χ***^**2**^	***P *value**
Type I	32 (58.2)	23 (41.8)	0.743	0.154	39 (70.9)	16 (29.1)	1.142	0.565
Type II	24 (80.0)	6 (20.0)			24 (80.0)	6 (20.0)		
Type III	4 (80.0)	1 (20.0)			4 (80.0)	1 (20.0)		

^a^χ^2^ test. ^b^Kruskal-Wallis test.

**Table 4:** Relationship between types of residual tumors and the expression changes

**Receptor**	**Change**	**Type of residual tumor**			
		**Type I**	**Type II**	**Type III**	***P *value**^**a**^
ER	Increase	2	2	0	0.839
	No change	41	20	4	
	Decrease	12	8	1	
PR	Increase	6	3	0	0.891
	No change	36	20	4	
	Decrease	13	7	1	
HER2	Increase	6	2	1	0.545
	No change	36	22	4	
	Decrease	13	6	0	

^a^Jonckheere-Terpstra test.

## Discussion

It has been shown that patients who have undergone modified radical mastectomy have a long-term survival rate similar to that of those who have undergone radical mastectomy [10-13]. NCT can make 23% of primary breast cancers that are otherwise unfit for BCS manageable with BCS through downstaging [4]. Wolmark et al. [3] reported a local recurrence rate of 10.7% in postchemotherapy patients treated with BCS, 7.6% in patients with primary tumors fit for BCS and 15.9% in patients with primary breast cancer unfit for BCS through downstaging by chemotherapy. The local recurrences were found mostly around the site of operation.

MRI observations of tumor morphological changes after NCT have been classified by contraction patterns into concentric contraction and patchlike contraction [2]. About 70% of residual tumors were previously found to be solitary and 30% were patchlike [5]. A negative surgical margin of BCS is closely associated with local recurrence-free and metastasis-free survival [3,4]. It was difficult to ensure negative surgical margins in BCS patients who had multifocal residual tumors, which was one of the factors that caused local recurrence [14,15]. However, some patients with solitary residual tumors that had negative surgical margins still developed local recurrence. There might be microfoci that are missed by conventional histological sections due to sample selection but could be revealed by subserial sectioning of the whole breast.

Successive breast subsections were mostly used to study multifocal primary breast cancer lesions, the scattering mode of breast cancer, Paget’s disease of the breast and diagnosis of occult breast cancer [16-18]. We used subserial sectioning of the whole breast to study the morphology of residual breast cancer and the changes in the tumor bed. The morphology of residual tumors after NCT observed by pathological examination was similar to the shrink patterns examined by imaging. Besides the two previously reported types of residual breast cancer after NCT, for example, solitary, multifocal and patchlike lesions, a third type of residual tumor which has one main cancer lesion and one or two small satellite lesions at least 1.0 cm away from it was also observed. As the satellite lesions are located away from the main residual tumor, the surgical margin may appear negative when examined by intraoperative frozen biopsy, but remaining satellite lesions could cause local recurrence.

The pattern of residual tumor is thought to relate to primary tumor size, and multifocal patchlike lesions more frequently result from larger primary tumors [19]. We have shown that the larger the primary tumors, the more types II and III residual tumors occurred, which may be due to vascular distribution and avascular necrosis. Age and menstrual status had no bearing on the pattern of residual tumors.

Chagpar et al. suggested that ultrasound and X-ray examinations cannot identify residual tumors and chemotherapy-related fibrotic changes, as X-ray underestimates the volume of larger tumors and ultrasound underestimates all breast lumps [20]. We detected nine cases of calcification with X-ray examination after NCT. Pathological examination showed that five of the nine cases of calcification had an intraductal cancer component, and four showed small blood vessel changes within the cancer or necrosis around blood vessels. For type I residual tumors, there was no difference in tumor size measured by ultrasound and pathological examination. However, there was a significant size difference between measurement by ultrasound and pathological examination of types II and III residual tumors. Ultrasound may not have enough sensitivity to penetrate the larger size of types II and III tumors.

It has been shown that the efficacy of NCT may be influenced by ER, PR and HER2 status [21-23]. The response to chemotherapy was different with regard to the types of cancer and drugs involved. Patients with triple-negative breast cancers had better responses to NCT and better long-term survival [22]. We looked at the possible correlation between ER, PR and HER2 status with the types of residual tumors after NCT. The levels of or the change of levels of ER, PR and HER2 were similar among cancers with different types of residual tumors after NCT, indicating that the impact of type II and type III on local recurrence and survival rate was not related to ER, PR or HER2 status.

## Conclusion

Patients with solitary lesions after chemotherapy were suitable for BCS, whereas the ones with multifocal and patchlike residual lesions required extra caution. BCS negative margins of type III residual tumors obtained from intraoperative frozen biopsies might miss satellite lesions and lead to local recurrence.

## Abbreviations

BCS: Breast conservation therapy; ER: Estrogen receptor; HER2: Human epidermal growth factor receptor 2; OS: Overall survival; PR: Progesterone receptor; RECIST: Response evaluation criteria in solid tumors; TE: Taxol and epirubicin.

## Competing interests

The authors declare that they have no competing interests.

## Authors’ contributions

SW, XY, LE, XQ and QC carried out data acquisition. YZ and JJ conceived the project and designed the study. SW, YZ and JJ drafted the manuscript. XQ and JJ carried out statistical analyses. All authors read and approved the final manuscript.
